# *Purpureocillium lilacinum*: Atypischer Erreger einer mykotischen Keratitis bei einer immunkompetenten Patientin

**DOI:** 10.1007/s00347-021-01325-4

**Published:** 2021-02-13

**Authors:** Loïc Hamon, Mohammed El Halabi, Fidelis A. Flockerzi, Berthold Seitz, Loay Daas

**Affiliations:** 1 Klinik für Augenheilkunde, Universitätsklinikum des Saarlandes (UKS), Kirrberger Str. 100, Gebäude 22, 66421 Homburg/Saar, Deutschland; 2 Institut für Allgemeine und Spezielle Pathologie, Universitätsklinikum des Saarlandes (UKS), Homburg/Saar, Deutschland

## Anamnese

Eine 73-jährige Patientin stellte sich notfallmäßig aufgrund seit einigen Tagen bestehender Augenschmerzen und zunehmender Visusminderung am linken Auge in unserer Klinik vor. Die Patientin hatte bis auf eine mit Brille gut korrigierte Hyperopie keine ophthalmologischen und systemischen Vorerkrankungen. Anamnestisch verneinte sie ein Trauma oder eine Verletzung am betroffenen linken Auge. Die Patientin trug keine Kontaktlinsen und war seit mehreren Jahren Rentnerin.

## Befunde

**Figure 1:**
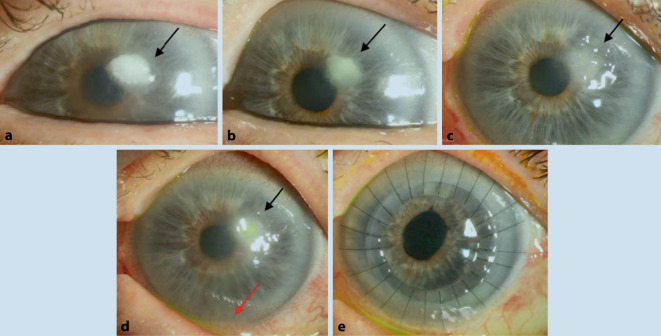

**Figure 2:**
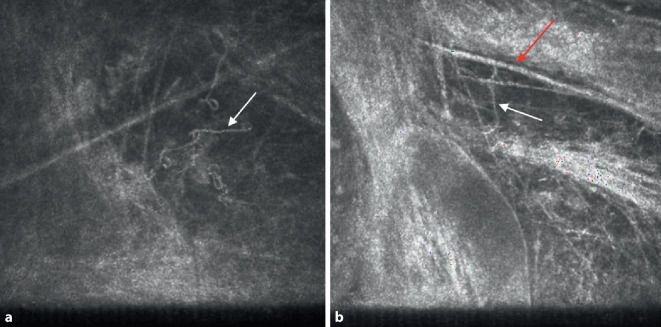

Der Visus betrug am Tag der Erstvorstellung bestkorrigiert (RA: +6,75/−0,75/A 26°; LA: +8,00/−2,00/A 117°) am rechten Auge 1,0 (nach Snellen) und am betroffenen linken Auge 0,5. Es zeigte sich spaltlampenbiomikroskopisch ein weiß-gräuliches, großflächiges, wolkenförmiges, unscharf begrenztes Hornhautinfiltrat (Größe ca. 1,5 × 1,0 mm), das sich bis tief ins Stroma ausbreitete, ohne Satellitenkonfiguration (Abb. 1a). Der restliche Vorderabschnitt war – bis auf eine mäßige Cataracta corticonuclearis – unauffällig. Es gab weder Hinweise auf ein Hypopyon noch auf eine Endophthalmitis. Am rechten Partnerauge ergab die Spaltlampenbiomikroskopie einen regelrechten, altersentsprechend unauffälligen Befund. In der konfokalen Mikroskopie zeigte sich bis zum mittleren Stroma ein dichtes Infiltrat mit am Rand serpiginösen, wurmförmigen, hyperreflektiven Strukturen, die typisch für einen filamentösen Pilz erschienen ([4]; Abb. 2).

## Diagnose

Ein diagnostisches Hornhautabradat wurde nach in vivo konfokalmikroskopischer Diagnose durchgeführt. Die mikrobiologische Untersuchung des Hornhautabradats (Kultur in einer Nährbouillon) ergab 10 Tage nach klinischer Erstdiagnose ein kulturelles Wachstum eines filamentösen Pilzes. Dieser wurde 4 Wochen nach Einreichung des Abradats als *Purpureocillium lilacinum* molekularbiologisch charakterisiert. Eine Resistenztestung wurde im Nationalen Referenzzentrum für Invasive Pilzinfektionen (NRZMyk) in Jena durchgeführt und bewies eine Sensibilität des Erregers für Voriconazol 0,125 mg/l, Posaconazol 0,06 mg/l, Itraconazol > 8 mg/ml, Isavuconazol 0,25 mg/l, Terbinafin 0,25 mg/l und Amphotericin B > 16 mg/l. Es ergab sich zusätzlich kein Wachstum eines anderen Pilzes oder einer Bakterienspezies.

## Therapie und Verlauf

Am Tag der klinischen in vivo konfokalmikroskopischen Diagnose wurde nach therapeutischer Hornhautabrasio eine Therapie mit Voriconazol 2 % Augentropfen (AT), Moxifloxacin AT und einem Kombipräparat von Polymyxin B + Neomycin + Gramicidin AT (Polyspectran® [Alcon Pharma GmbH, Lebach, Deutschland]) stündlich im Wechsel begonnen. Nach 2 Wochen zeigte sich eine Befundbesserung mit beginnender Abgrenzung des Infiltrats (Abb. 1b). Bei Persistenz des Infiltrats wurden aufgrund der Sensibilität in der Resistenztestung zusätzlich Amphotericin B 2 % AT, Polyhexanid AT und Voriconazol oral (200 mg 2‑mal pro Tag) zusätzlich appliziert, nachdem bei fehlender Rückbildung eine lamelläre Keratektomie im Bereich des Infiltrats mit Mini-Trepan (3,5 mm) erfolgt war. Danach wurde die Patientin mit Reduktion der lokalen Therapie von halbstündlich im Wechsel auf eine 5‑malige Gabe pro Tag entlassen (Abb. 1c). Vier Wochen danach zeigte sich eine Verschlechterung des Hornhautbefundes in Form einer beginnenden Hypopyonbildung links (Abb. 1d). Nach erneuter stationärer Aufnahme führten wir eine intrakamerale Eingabe von Voriconazol, Amphotericin B und Ceftazidim („Medikamenteneingabe“) durch und intensivierten die Therapie mit Voriconazol 2 % AT, Polyhexanid AT, Polyspectran® und Moxifloxacin AT stündlich im Wechsel sowie Voriconazol 200 mg oral 2‑mal pro Tag und – aufgrund seines synergistischen Effekts mit Voriconazol [9] – Terbinafin oral 250 mg pro Tag. Wir führten nach 3 Tagen unter oben genannter konservativer Therapie eine perforierende Excimerlaser-Keratoplastik à chaud (8,5/8,6 mm) mit Phakoemulsifikation der Linse und Implantation einer Hinterkammerlinse (sog. „Triple-Prozedur“) mit zusätzlicher intrakameraler Medikamenteneingabe durch (Abb. 1e). Bei der Entlassung wurde die lokale Therapie auf je 5‑mal pro Tag reduziert. Als Alternative zu den üblichen Kortikosteroiden, die früh postoperativ aufgrund des Risikos eines deletären Verlaufs nicht gegeben werden sollten [10], wurde initial postoperativ die 2‑malige Gabe pro Tag von Ciclosporin A 0,1 % AT und Ciclosporin A 150 mg per os als lokale und systemische immunsupprimierende Therapie eingesetzt. Vier Wochen nach Entlassung zeigte sich weder auf dem Transplantat noch auf dem Wirtsgewebe ein Rezidiv der mykotischen Keratitis (Abb. 1e). Der Visus betrug unkorrigiert 1/7,5 (Lesetafel) am betroffenen Auge. Die lokale Therapie zu diesem Zeitpunkt bestand aus Voriconazol 2 % AT 5‑mal/Tag (für 2 Monate), Ciclosporin A 0,1 % AT 2‑mal/Tag und Prednisolonacetat AT 10 mg/ml 5‑mal/Tag (Reduktion von 1 Tropfen alle 8 Wochen bis 3‑mal/Tag – dann bei 3‑mal/Tag belassen), welche 1 Woche nach der Keratoplastik aufgrund des regelrechten Befundes begonnen wurde. Systemisch wurden am Tag der Untersuchung Ciclosporin A und Voriconazol bei erhöhten Leberwerten abgesetzt.

**Figure 3:**
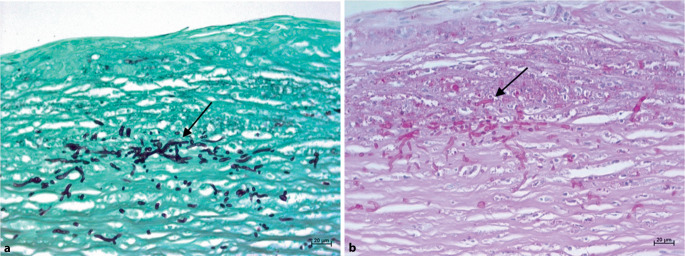

Die Diagnose einer mykotischen Keratitis wurde postoperativ (sowohl im lamellären als auch im größeren perforierenden Exzisat) mit Nachweis von Pilzhyphen im anterioren Stroma des Hornhautexzisates histologisch bestätigt (Abb. 3).

## Diskussion

In den Industrienationen sind mykologische Organismen ursächlich für 1–5 % der infektiösen Keratitiden [14]. Diese Erreger können einen fulminanten oder aber auch einen protrahierten und rezidivierenden Verlauf zeigen [3, 15]. Anhand des deutschen Pilz-Keratitis-Registers wurden zwischen 2000 und 2017 als häufigste Erreger einer Pilzkeratitis *Fusarium spp.* (36,7 %), *Candida spp.* (35,8 %) und *Aspergillus spp.* (6,4 %) genannt. Zu den anderen, deutlich selteneren Pilzen mit Augentropismus zählt *Purpureocillium lilacinum* [12].

*Purpureocillium lilacinum* (früher *Paecilomyces lilacinus*) ist ein saprophytischer Fadenpilz der Familie *Ophiocordycipitaceae*. Er besiedelt kultivierte und nicht kultivierte Böden sowie Pflanzenmaterial aufgrund seiner Anwendung in der Landwirtschaft als Biokontrollmittel gegen Nematoden. Risikofaktoren für eine mögliche Infektion sind ein reduzierter Immunstatus (z. B. immunsupprimierende Therapie), berufliche Exposition (z. B. Gartenarbeit oder Landwirtschaft) und das Tragen von Kontaktlinsen [3]. Sehr selten kann es auch nach Laser-in-situ-Keratomileusis (LASIK) zu einer Pilzkeratitis im Interface kommen [7]. Aufgrund der Produktion von hydrolytischen Enzymen kann dieser Pilz intakte Membranen penetrieren und führt vornehmlich zur Durchwanderungsendophthalmitis bei intaktem Epithel [15]. Bei unserer Patientin konnte keiner dieser Risikofaktoren erhoben werden. Die Patientin war nicht immunsupprimiert, trug keine Kontaktlinse und arbeitete nicht in der Landwirtschaft. Sie besaß ein Haus mit Garten, wo sie sich anamnestisch oft aufhielt. Aus diesem Grund ist diese opportunistische Infektion hier besonders atypisch.

Im Frühstadium kann eine mykotische Keratitis klinisch nicht sicher von anderen mikrobiellen Keratitiden unterschieden werden [6]. Da eine verzögerte Diagnose die Prognose einer visuellen Erholung deutlich reduzieren kann, spielen zur frühzeitigen Differenzialdiagnose paraklinische Untersuchungen eine wichtige Rolle, hier v. a. die in vivo konfokale Mikroskopie. Diese ermöglicht die nichtinvasive Diagnose einer mykotischen Keratitis am Tag der Aufnahme. Sie erreicht eine Sensitivität von 80–90 % und liegt damit über der Sensitivität der Erregeridentifizierung durch mikrobiologische Kulturen [4]. Eine assoziierte Hornhaut-Endothel-Epithel-Dekompensation kann jedoch die Sensitivität bei tiefstromalem oder endothelialem Infiltrat deutlich reduzieren und dadurch die Diagnose vor einer möglichen Keratoplastik nicht unterstützen. In solchen Fällen kann alternativ eine postkeratoplastische invertierte in vitro konfokale Mikroskopie am Tag der Keratoplastik zur Diagnosebestätigung vor histologischem und mikrobiologischem Ergebnis durchgeführt werden [5]. Direktausstrich von gewonnenem Material (z. B. Hornhautabradat) ermöglicht einen schnellen, aber untersucher- und materialabhängigen Nachweis des Erregers bei mykotischer Keratitis, hierbei jedoch mit deutlicher Einschränkung einer möglichen Differenzierung der Spezies [2]. Eine Hornhautabrasio zur Gewinnung von Material für eine mikrobiologische Kultur sowie weitere molekularbiologische Analysen (Polymerasekettenreaktion [PCR]) sollte am Tag der Aufnahme durchgeführt werden. Unter anderem durch molekularbiologische Techniken wie die Sequenzierung der „Internally transcribed spacer-2“(ITS-2)-Region kann eine rasche Charakterisierung der Pilzspezies gelingen und in der Folge ein geeignetes Antimykotikum eingesetzt werden [13].

Es gibt derzeit keine Leitlinien für die antimykotische Behandlung von *Purpureocillium-lilacinum*-Keratitis. Amphotericin B, Natamycin, Fluconazol, Echinocandine und Flucytosin sind im Allgemeinen unwirksam [3, 15]. Im Gegensatz dazu zeigten die Triazole einen wirksamen therapeutischen Effekt, hierbei stellt sich Voriconazol als wirksamstes Agens vor [3]. Ein Synergieeffekt gegen Pilzhyphen einschließlich gegen *Purpureocillium lilacinum* wurde mit Triazolen und Terbinafin beschrieben, sodass die Kombination von topischem Voriconazol sowie oralem Terbinafin, wie es auch im beschriebenen Fall erfolgreich angewendet wurde, aktuell die aussichtsreichste Therapie ist [9]. Diese kann mit oralem Voriconazol ergänzt werden [3], jedoch ohne Beweise einer höheren Effizienz [11]. Bei Resistenz gegen Voriconazol wurde Posaconazol oral in Kombination mit einer perforierenden Keratoplastik bei einem Patienten aus Portugal erfolgreich eingesetzt [1].

Fadenpilze zeigen häufig einen fulminanten oder rezidivierenden Verlauf und können oft, wie auch bei unserer Patientin, neben einer konservativen Therapie eine perforierende Keratoplastik à chaud benötigen [2, 8]. Bei diesen Fällen sollten lokale und systemische Kortikosteroide erst verzögert eingesetzt werden. Diese können ansonsten während der postoperativen Frühphase zu einem deletären Verlauf führen [10]. Als Alternative bieten sich nichtsteroidale Immunsuppressiva wie Ciclosporin A 0,05 bis 2 % AT an [2, 10].

## Fazit für die Praxis

- *Purpureocillium lilacinum* ist ein seltener Erreger einer mykotischen Keratitis.
- Risikofaktoren für eine *Purpureocillium-lilacinum*-assoziierte Keratitis sind ein reduzierter Immunstatus, das Tragen von Kontaktlinsen sowie der Kontakt mit Pflanzenmaterial.
- Mithilfe der in vivo konfokalen Mikroskopie kann auch bei untypischem klinischem Bild noch vor dem mikrobiologischen Nachweis des Erregers eine frühzeitige Diagnose einer Pilzkeratitis gestellt werden.
- Eine rechtzeitige antimykotische lokale und ggf. systemische Therapie mit Voriconazol bietet die besten Heilungschancen. Eine adjuvante Einnahme von Terbinafin ist aufgrund seines synergetischen Effekts mit Triazolen indiziert.
- Filamentöse Pilze haben oft einen fulminanten oder rezidivierenden Verlauf und benötigen nicht selten trotz medikamentöser Therapie eine perforierende Keratoplastik à chaud, dann postoperativ mit verzögerter Kortikosteroidapplikation.
